# Assessment of Pendelluft Using Electrical Impedance Tomography in a Patient With Flail Chest

**DOI:** 10.1002/rcr2.70583

**Published:** 2026-04-09

**Authors:** Naruaki Otake, Hidefumi Sano, Jushi Numata, Takeo Nagura, Junya Tsurukiri

**Affiliations:** ^1^ Department of Emergency and Critical Care Medicine Tokyo Medical University Hachioji Medical Center Tokyo Japan

**Keywords:** EIT, flail chest, Pendelluft

## Abstract

An 80‐year‐old man with flail chest exhibiting paradoxical chest wall movement required mechanical ventilation for internal fixation. Despite the apparent paradoxical motion resolution, unstable chest wall segments allowed pendelluft to persist. Thus, EIT‐guided management of flail chest should focus on stabilising the visible chest wall and achieving homogenised ventilation dynamics.

## Graphical abstract

This case illustrates that EIT‐guided management of flail chest should focus on stabilising the visible chest wall and achieving homogenised ventilation dynamics.

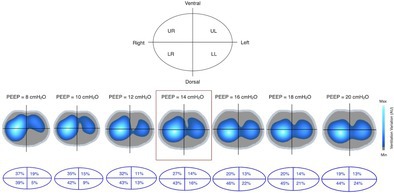

An 80‐year‐old man with multiple rib fractures and flail chest exhibiting paradoxical chest wall movement required mechanical ventilation due to tachypnea (24 breaths/min) and increased work of breathing (Figure 1). Pressure‐controlled ventilation was initiated to stabilise the chest wall (driving‐pressure 11 cmH_2_O, positive end‐expiratory pressure [PEEP] 8 cmH_2_O and FiO_2_ 60%) and paradoxical movement was no longer visible; however, electrical impedance tomography (EIT) revealed pendelluft, characterised by intrapulmonary airflow from nondependent to dependent regions during a single breath without a substantial change in tidal volume. EIT‐based analysis of regions of interest (ROIs) showed that ventilation distribution expanded from ventral to dorsal and right to left as PEEP increased. During PEEP titration (from 5 cmH_2_O to 20 cmH_2_O), pendelluft was markedly reduced at PEEP 14 cmH_2_O, accompanied by improvements in P0.1 (from −3.4 cmH_2_O to −2.1 cmH_2_O) and respiratory system compliance (Crs, from 60 mL/cmH_2_O to 80 mL/cmH_2_O) (Figures 2 and 3). Arterial blood gas analysis showed improvements in pH (7.316–7.326), PaO_2_ (109–130 mmHg) and PaCO_2_ (33.1–29.3 mmHg), accompanied by a reduction in respiratory rate to approximately 15 breaths/min. However, Crs decreased at PEEP ≥ 16 cmH_2_O.

**FIGURE 1:**
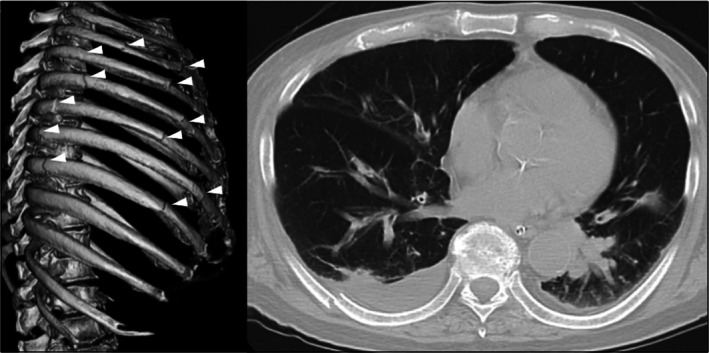
Chest computed tomography images showing multiple rib fractures (arrowhead) and areas of atelectasis.

**FIGURE 2:**
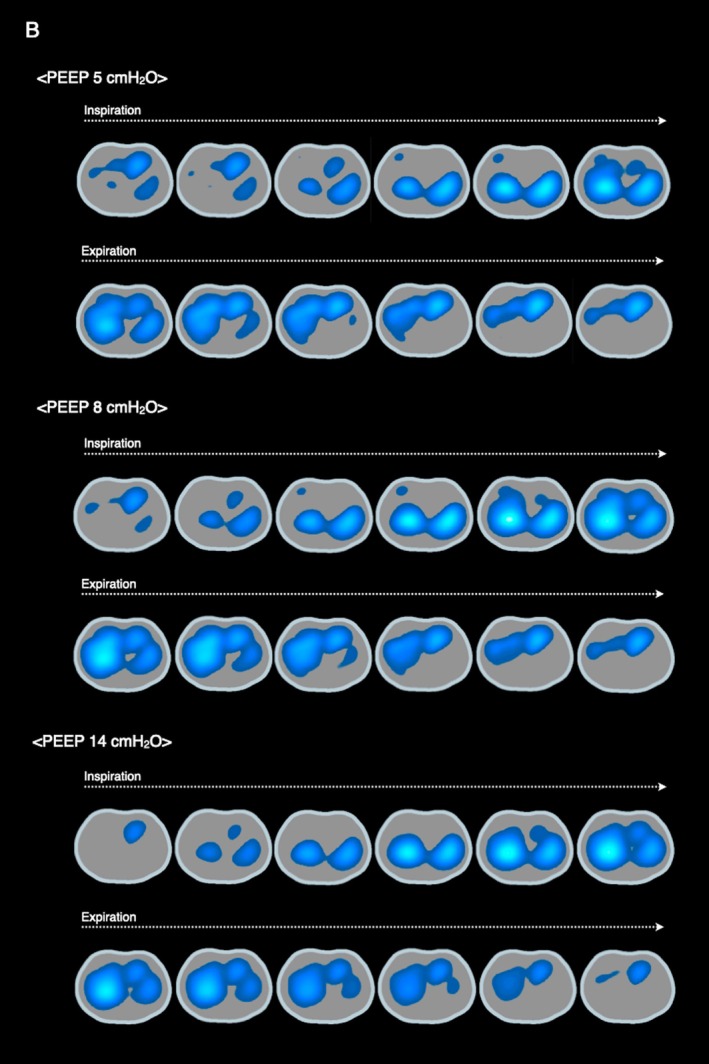
At a positive end‐expiratory pressure (PEEP) of 5 cmH_2_O, early inflation of the dorsal regions during inspiration and delayed inflation of the ventral regions during expiration indicate the presence of pendelluft. Additionally, heterogeneous inflation in the right ventral regions is observed during early inspiration. At a PEEP of 8 cmH_2_O, pendelluft, characterised by gas movement from the dorsal to the ventral lung regions, remains evident along with persistent heterogeneous inflation in the right ventral regions. At a PEEP of 14 cm H_2_O, heterogeneous inflation resolved, and ventilation distribution improved in the dorsal–ventral and lateral directions.

**FIGURE 3:**
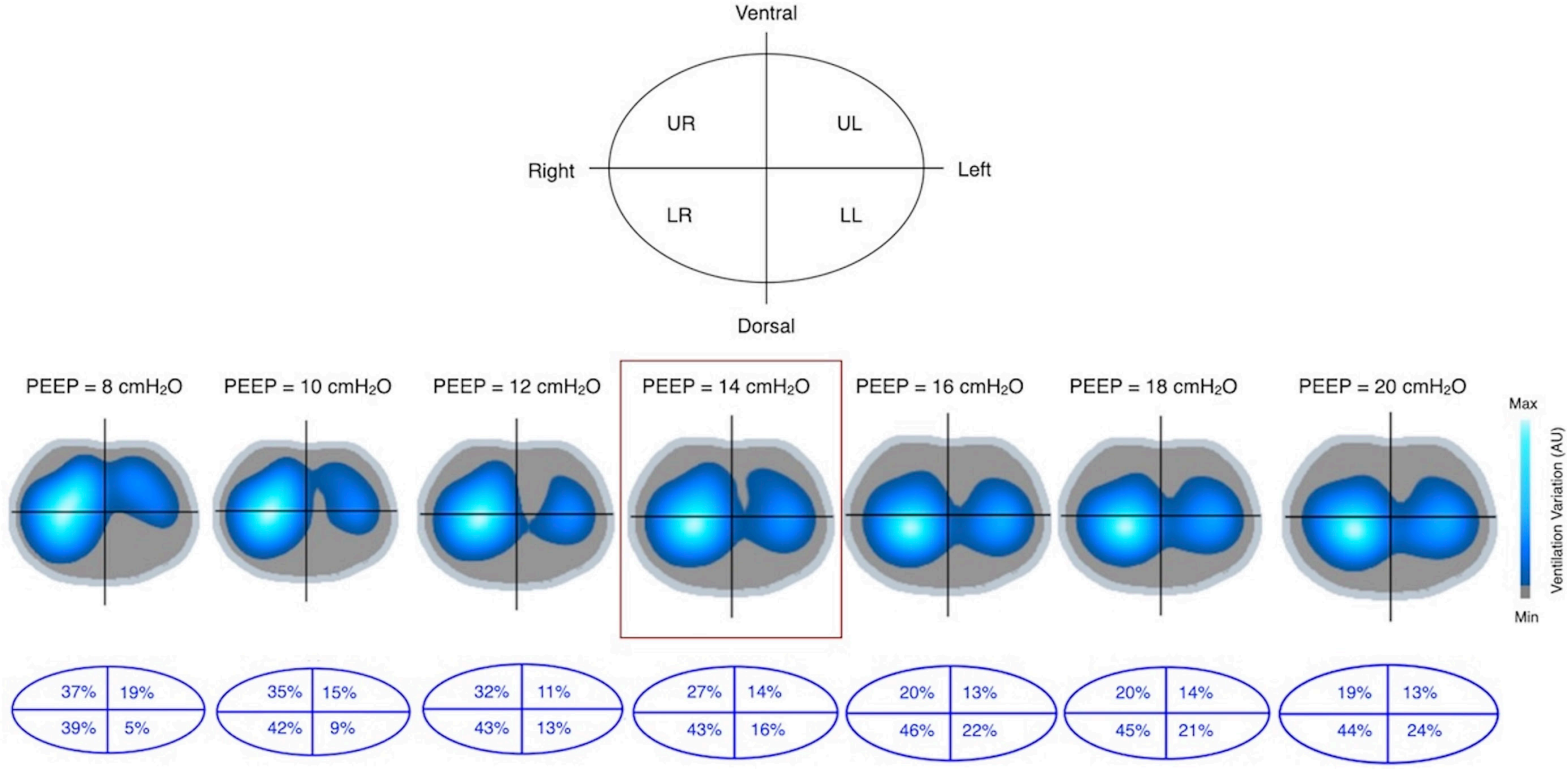
To assess regional ventilation distribution across regions of interest (ROIs), tidal impedance variation images were divided into four equal quadrants: Upper right, upper left, lower right and lower left. Ventilation distribution, expressed as a percentage of total lung ventilation, expanded from dorsal to ventral and from right to left as PEEP increases. AU, arbitrary units; LL, lower left; LR, lower right; PEEP, positive end‐expiratory pressure; UL, upper left; UR, upper right.

Pendelluft may arise from regional differences in time constants or dynamic pleural‐pressure swings during spontaneous breathing and may promote regional overstretch and lung injury [1]. In patients with flail chest, unstable chest wall segments may disrupt local pleural‐pressure transmission and interact with parenchymal heterogeneity due to contusion or haemothorax, producing regional pressure gradients that sustain pendelluft even after paradoxical motion resolution [2]. These findings suggest that EIT‐based management of flail chest should aim to achieve visible chest wall stabilisation and ensure ventilation dynamics homogenisation.

## Author Contributions

N.O. and J.T. conceived and designed the study. H.S., J.N. and T.N. provided technical support.

## Funding

The authors have nothing to report.

## Consent

The authors declare that written informed consent was obtained for the publication of this manuscript and accompanying images using the consent form provided by the Journal.

## Conflicts of Interest

The authors declare no conflicts of interest.

## Data Availability

Research data are not shared.
